# Hotspots and research trends of gut microbiome in polycystic ovary syndrome: a bibliometric analysis (2012–2023)

**DOI:** 10.3389/fmicb.2024.1524521

**Published:** 2025-01-08

**Authors:** Ruishan Wu, Zhensheng Mai, Xiaoyan Song, Wenzhong Zhao

**Affiliations:** ^1^NHC Key Laboratory of Male Reproduction and Genetics, Guangdong Provincial Reproductive Science Institute (Guangdong Provincial Fertility Hospital), Guangzhou, China; ^2^Department of Obstetrics and Gynecology, First People’s Hospital of Foshan, Foshan, China

**Keywords:** gut microbiome, polycystic ovary syndrome (PCOS), bibliometric, pathogenesis, treatment, CiteSpace, VOSviewer

## Abstract

**Introduction:**

Polycystic ovary syndrome (PCOS) is a common gynecological condition affecting individuals of reproductive age and is linked to the gut microbiome. This study aimed to identify the hotspots and research trends within the domain of the gut microbiome in PCOS through bibliometric analysis.

**Methods:**

Utilizing bibliometric techniques, we examined the literature on the gut microbiome in PCOS from the Web of Science Core Collection spanning the period from 2012 to 2023. Analytical tools such as CiteSpace, VOSviewer, and Bibliometric R packages were employed to evaluate various metrics, including countries/regions, institutions, authors, co-cited authors, authors’ H-index, journals, co-references, and keywords.

**Results:**

A total of 191 publications were identified in the field of gut microbiome in PCOS, with an increase in annual publications from 2018 to 2023. People’s Republic of China was the most productive country, followed by the United States of America (USA), India. Shanghai Jiao Tong University, Fudan University, and Beijing University of Chinese Medicine were the top three most publications institutions. Thackray VG was identified as the most prolific author, holding the highest H-index, while Liu R received the highest total number of citations. The journal “Frontiers in Endocrinology” published the most articles in this domain. The most frequently co-cited reference was authored by Qi XY. The analysis of keyword burst detection identified “bile acids” (2021–2023) as the leading frontier keyword. Additionally, “gut dysbiosis,” “phenotypes,” “adolescents,” “metabolomics,” “metabolites,” “fecal microbiota transplantation,” and “IL-22” have emerged as the primary keywords reflecting recent research trends.

**Conclusion:**

This bibliometric analysis explores how the gut microbiome influences endocrine and metabolic disorders related to PCOS, emphasizing its role in the development of PCOS and treatments targeting the gut microbiome. The findings serve as a valuable resource for researchers, enabling them to identify critical hotspots and emerging areas of investigation in this field.

## 1 Introduction

Polycystic ovary syndrome (PCOS) is a multifactorial, polygenic, and complex endocrine disorder prevalent among females of reproductive age, with a global prevalence ranging from 5% to 15% (McCartney and Marshall, 2016; Wang et al., 2019; Dapas and Dunaif, 2022). PCOS is characterized by a spectrum of interrelated reproductive abnormalities, including dysregulated gonadotropin secretion, hyperandrogenism, chronic anovulation, and polycystic ovarian morphology (Dapas and Dunaif, 2022). It is frequently associated with major causes of infertility, insulin resistance, and obesity (Yurtdas and Akdevelioglu, 2020; Dapas and Dunaif, 2022; Rezaei-Golmisheh et al., 2024). Despite extensive research over several decades, the etiology of PCOS remains largely unknown (Dapas and Dunaif, 2022). Compared with polycystic ovarian disease with only polycystic ovarian morphology which lacked the other diagnostic criteria of PCOS, the symptoms and treatment of PCOS are more complex (Torres et al., 2018). Simple polycystic ovarian morphology may present only as irregular menstruation or occasional ovulation disorder but can be improved by adjusting the lifestyle (Moran et al., 2011; Abdulkhalikova et al., 2022). In contrast, PCOS treatment may involve lifestyle adjustments, medication, surgery, or *in vitro* fertilization and embryo transfer for those with persistent ovulation problems or additional infertility factors (Thessaloniki, 2008).

The gut microbiome is increasingly acknowledged as an endocrine organ due to its production of metabolites that exert direct or indirect effects on the host’s physiological processes (Tang et al., 2019). These influences extend to the host’s immune response, lipid profiles, neural functions, energy homeostasis, and glucose metabolism (Tang et al., 2019; de Vos et al., 2022). Contemporary research has elucidated a significant association between the gut microbiome and a spectrum of diseases, including atherosclerosis, hypertension, heart failure, chronic kidney disease, obesity, type 2 diabetes mellitus, Alzheimer’s disease, gastrointestinal cancers, hepatic disorders, and so on (Tang et al., 2019; Trebicka et al., 2021; Yoo et al., 2021; de Vos et al., 2022; Chandra et al., 2023; Kim et al., 2024). Alterations in the gut microbiome have been linked to female infertility disorders, including PCOS, endometriosis, and premature ovarian failure. Dysbiosis of the gut microbiota may directly or indirectly contribute to the pathogenesis of these infertility disorders (Wang et al., 2024). Recently, there has been an increasing scholarly interest in the relationship between the gut microbiome and PCOS, leading to a proliferation of studies on this topic.

Bibliometric analysis has been employed to conduct both quantitative and qualitative evaluations of literature within the domains of mathematics and statistics. This approach facilitates a systematic visualization of the evolution of research topics, thereby highlighting current trends and focal areas within a given field (Yang et al., 2022). Furthermore, bibliometric methods offer a more objective means of analysis (Hicks et al., 2015). These methods synthesize extensive reference data to provide a comprehensive overview of the current state of knowledge (Solmi et al., 2022). They facilitate the identification of research abundances, gaps, and trends, while also elucidating potential moderators, biases, and limitations within the research over time (Sabe et al., 2022; Solmi et al., 2022). Notable bibliometric analysis software includes Citespace, VOSviewer, and Bibliometric R, among others. By using bibliometric analysis software to create a knowledge map, a comprehensive literature review of the proposed research dimensions has been carried out (Liu et al., 2022).

At present, there is a lack of bibliometric analysis of the gut microbiome in PCOS. This study aims to conduct a bibliometric analysis of the literature on the gut microbiome in PCOS to find the relationship between the gut microbiome and PCOS and to identify the trends and hot topics in the field.

## 2 Materials and methods

### 2.1 Data source

The data for this analysis were sourced from the Science Citation Index Expanded (SCI-EXPANDED) within the Web of Science Core Collection. Through conducting a comprehensive literature search spanning from January 1, 2012, to December 31, 2023, to identify pertinent studies. The search utilized Medical Subject Headings terms and keywords, including “gut microbiome” and “polycystic ovary syndrome.” The complete search strategy is detailed in Supplementary information 1. To ensure the accuracy of the literature retrieved, the search was refined using the paper title (TI), abstract (AB), and author keyword (AK) fields within the Web of Science Core Collection topic module (Fu et al., 2012; Yang et al., 2022). Only original research articles and review articles published in English were included in this analysis while meeting abstracts, early access, editorial materials, corrections, and publications with expressions of concern were excluded. Complete records and cited references were stored in an unformatted manner.

### 2.2 Data analysis

**Figure 1:**
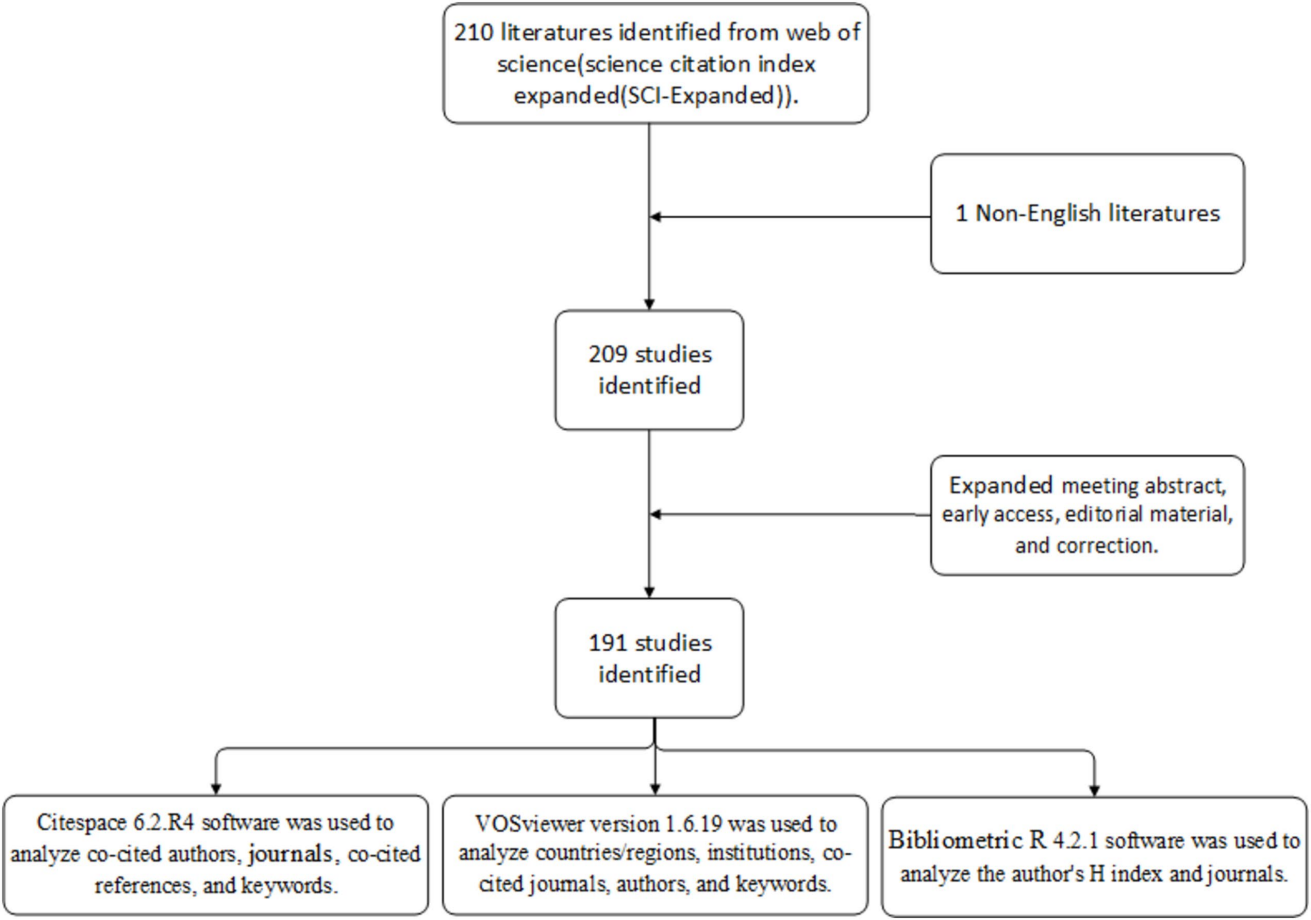
The flowchart for searching and analyzing the gut microbiome in PCOS.

Utilizing Citespace software (6.2.R4), VOSviewer software (1.6.19), and Bibliometric R (4.2.1), we performed a comprehensive visual analysis of the literature on met requirements. Additionally, Microsoft Office Excel 2021(Microsoft, Redmond, Washington, USA) was employed to illustrate publication trends in the field. Citespace serves as a bibliometric analysis tool adept at visualizing emerging trends and thematic shifts within the scientific literature, thereby offering valuable clinical evidence for researchers (Chen and Chen, 2005). When conducting a Citespace analysis, the criteria were satisfied with a Modularity *Q* value greater than 0.3 and a Mean Silhouette value exceeding 0.5. The period was segmented into annual intervals from January 2012 to December 2023. The selection criteria for the analysis of reference co-citation employed the g-index with a parameter of *k* = 25. VOSviewer, another bibliometric analysis software, is renowned for its proficiency in presenting extensive bibliometric maps in an accessible format (van Eck and Waltman, 2010). For the analysis of keywords using VOSviewer, a minimum occurrence threshold of four was established for the construction of collaboration networks. Bibliometric, programmed in R, is a flexible tool that can be quickly updated and integrated with other statistical R packages (Aria and Cuccurullo, 2017). Figure 1 shows the flowchart for search and analysis of the gut microbiome and PCOS.

## 3 Results

### 3.1 The trend of annual publication

**Figure 2:**
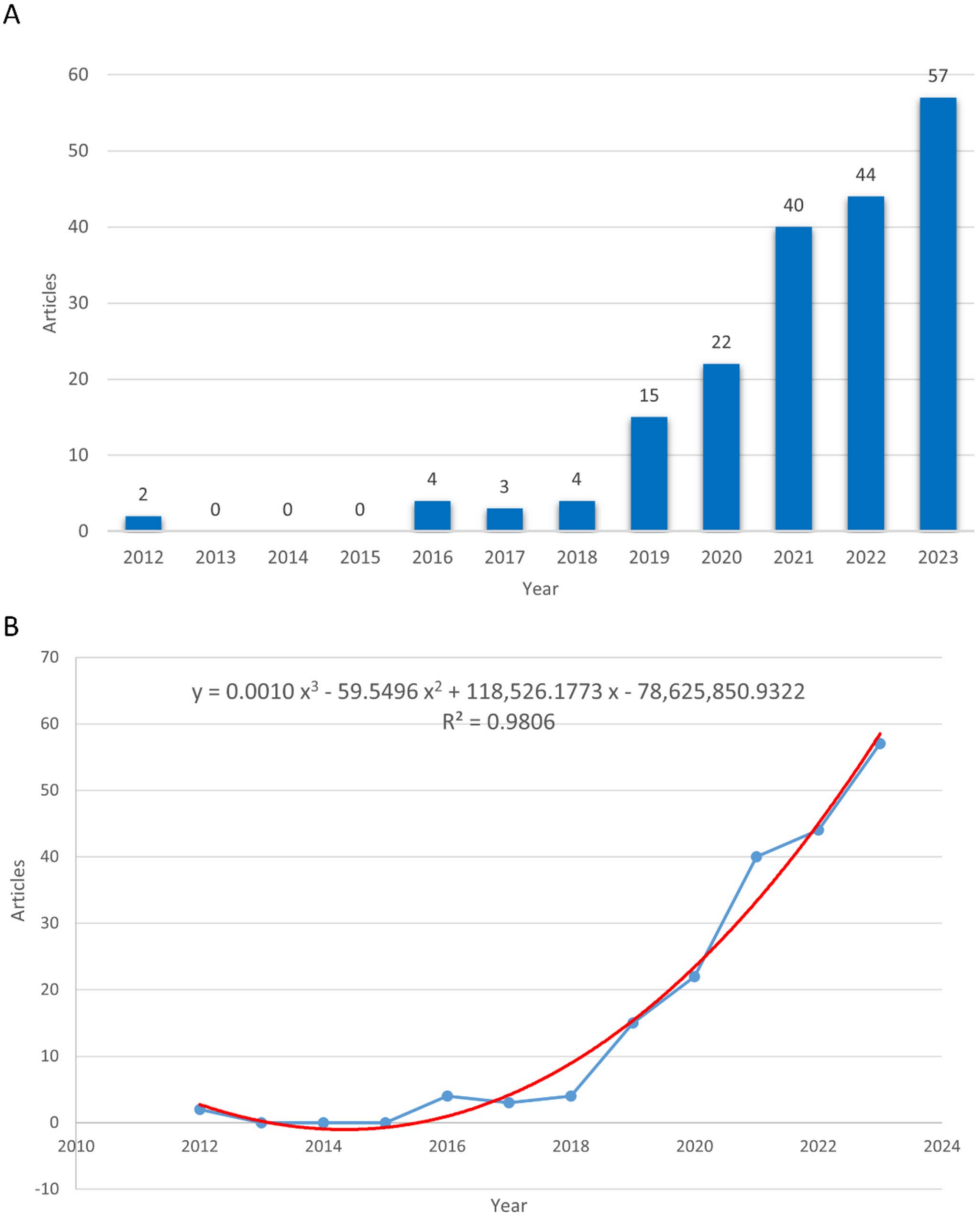
The number of publications and publication trends on gut microbiome in PCOS. **(A)** The number of publications in every year. **(B)** The publication trends in the field. The blue line represents the actual number of publications, while the red line indicates the forecasted number of publications.

A total of 191 publications on the gut microbiome in PCOS were identified between 2012 and 2023. Of these, 127 were original research articles (66.49%) and 64 were review articles (33.51%). As illustrated in Figure 2A, the period from 2012 to 2018 saw relatively few publications, followed by a consistent annual increase from 2019 to 2023. This trend suggests that research on the gut microbiome and PCOS was in its nascent stages during the earlier years. In 2012, there were merely two publications on the subject, which increased to 57 by 2023, indicating a growing academic interest in the study of the gut microbiome with PCOS. A polynomial regression model was built to forecast annual publication counts (R2 = 0.9806), the estimated number of publications by 2030 is approximately 211 (Figure 2B).

### 3.2 Countries/regions

A total of 37 countries/regions published articles on the topic of the gut microbiome in PCOS. The People’s Republic of China led with the highest number of publications (*N* = 115, 60.21%), followed by the United States of America (USA) (*N* = 28, 14.66%), India (*N* = 9, 4.71%), Italy (*N* = 9, 4.71%), and Türkiye (*N* = 7, 3.66%). China and the USA have emerged as the most influential contributors in this domain, as indicated by their leading positions in total citation counts. China occupies the first position with 2,737 citations, while the USA follows with 2,042 citations. Moreover, these two countries exhibit the highest total link strength in the field, with China achieving a total link strength of 13 and the USA attaining a total link strength of 17(Table 1).

**Table 1:** Top 10 countries/regions with the most publications on the gut microbiome in PCOS.

Rank	Country or region	Documents	Total citations	Total link strength	Average citations
1	China	115(60.21%)	2,737	13	23.80
2	USA	28(14.66%)	2,042	17	72.93
3	India	9(4.71%)	89	8	9.89
4	Italy	9(4.71%)	219	2	24.33
5	Türkiye	7(3.66%)	188	2	26.86
6	England	6(3.14%)	568	8	94.67
7	Iran	6(3.14%)	89	0	14.83
8	Poland	5(2.61%)	234	7	46.80
9	Australia	5(2.61%)	224	4	44.80
10	Austria	4(2.09%)	214	6	53.50

**Figure 3:**
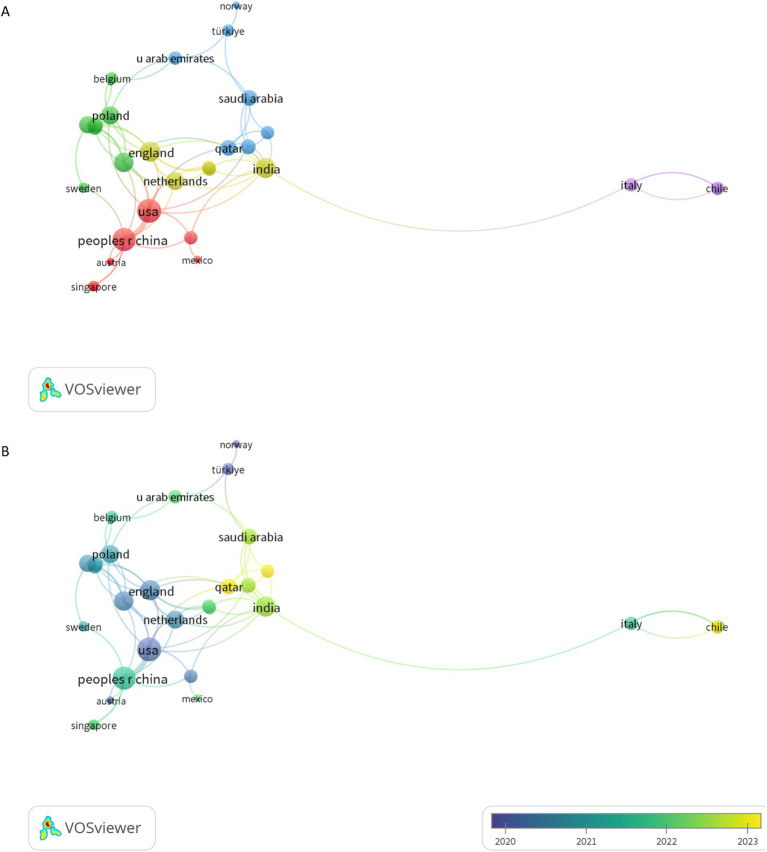
The visual analysis of co-authorship countries/regionals. **(A)** The co-authorship countries/regionals network visualization. **(B)** The co-authorship countries/regionals overlay visualization. The thicker the line the stronger cooperation.

Through VOSviewer analysis, China, the USA, Australia, Austria, Canada, England, the Netherlands, and Sweden exhibited cooperative relationships, with particularly robust collaboration between China and the USA (Figure 3A). The majority of these cooperative relationships among countries/regions were observed between 2020 and 2023 (Figure 3B).

### 3.3 Institutions

Through VOSviewer analysis, 333 institutions were identified as contributors to the body of literature on the gut microbiome in PCOS. The top 10 institutions, each with a minimum of five publications, were highlighted. Shanghai Jiao Tong University emerged as the leading institution with 11 publications, followed by Fudan University with 9, Beijing University of Chinese Medicine with 8, Shandong University with 7, and the University of California, San Diego, also with 7 (Table 2). The majority of these institutions were based in China. In terms of total citations, Peking University ranked highest with 610 citations, followed by the University of California, San Diego, with 523 citations, and San Diego State University with 427 citations.

**Table 2:** Top 10 institutions with the most publications on the gut microbiome in PCOS.

Rank	Institution	Publication	country	Total citations	Average citations
1	Shanghai Jiao Tong University	11	China	375	34.09
2	Fudan University	9	China	118	13.11
3	Beijing University of Chinese Medicine	8	China	91	11.38
4	Shandong University	7	China	130	18.57
5	University of California SanDiego	7	USA	523	74.71
6	Peking University	6	China	610	101.67
7	San Diego State University	6	USA	427	71.17
8	Shanghai Key Lab Assisted Reprod and Reprod Genet	5	China	126	25.20
9	Southern Medical University	5	China	64	12.80
10	Peking University Third Hospital	5	China	367	73.40

### 3.4 Authors and co-cited authors

**Figure 4:**
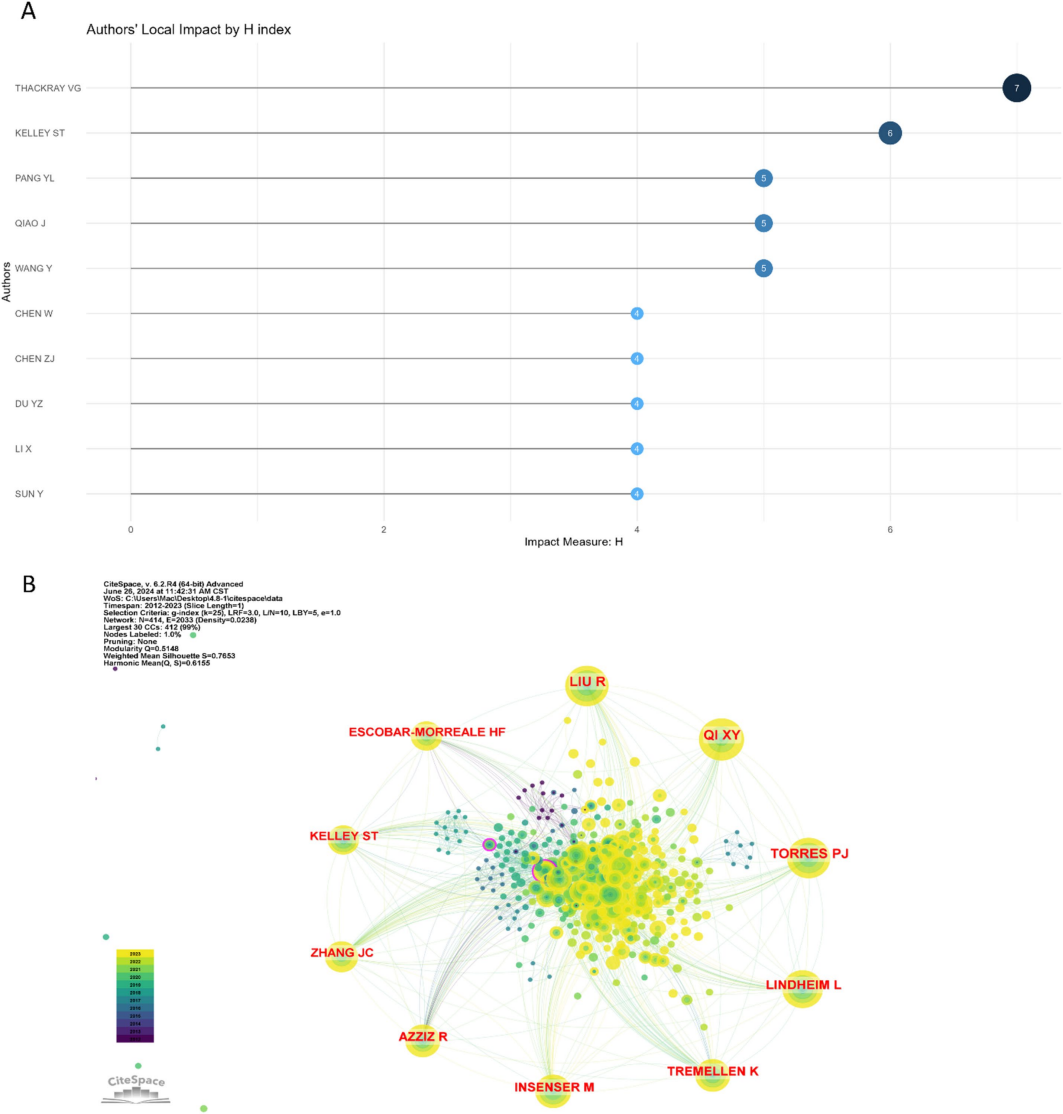
The authors’ local impact *H* index and co-cited authors on gut microbiome in PCOS. **(A)** The top 10 authors’ local impact *H* index in the field. **(B)** The top 10 co-cited authors on gut microbiome in PCOS, and the larger the circle, the more frequently it has been co-cited.

A total of 1,223 authors have contributed to the body of literature on the gut microbiome in PCOS. Table 3 enumerates the top 10 authors with the highest publication counts in this domain. Notably, Thackray VG, Kelley ST, Pang YL, and Qiao J emerged as the most prolific contributors. Among these, Thackray VG and Kelley ST demonstrated the highest local impact H-indexes, with values of 7 and 6, respectively (Figure 4A). Furthermore, both Thackray VG and Kelley ST were affiliated with institutions in the USA and exhibit significant scholarly interconnections within this research area.

**Table 3:** Top 10 authors and co-cited authors with the most publications on the gut microbiome in PCOS.

Rank	Author	document	Co-cited authors	citations
1	Thackray VG	7	Liu R	102
2	Kelley ST	6	Qi XY	99
3	Pang YL	5	Torres PJ	96
4	Qiao J	5	Lindheim L	88
5	Chen W	4	Insenser M	73
6	Chen ZJ	4	Tremellen K	66
7	Du YZ	4	Azziz R	65
8	Ni ZX	4	Zhang JC	59
9	Torres PJ	4	Kelley ST	55
10	Yu CQ	4	Escobar-morreale HF	55

Utilizing Citespace to analyze co-cited authors within the domain of gut microbiome and PCOS, Figure 4B demonstrates a Modularity Q value of 0.5148 and a Mean Silhouette score of 0.7653, thereby satisfying the analytical requirements. The top 10 co-cited authors, each cited at least 55 times, are illustrated in Figure 4B and detailed in Table 3. Notably, Liu R achieved the highest number of total citations (*n* = 102), followed by Qi XY (*n* = 99), Torres PJ (*n* = 96), Lindheim L (*n* = 88), and Insenser M (*n* = 73).

### 3.5 Journal and co-cited journal analysis

**Figure 5:**
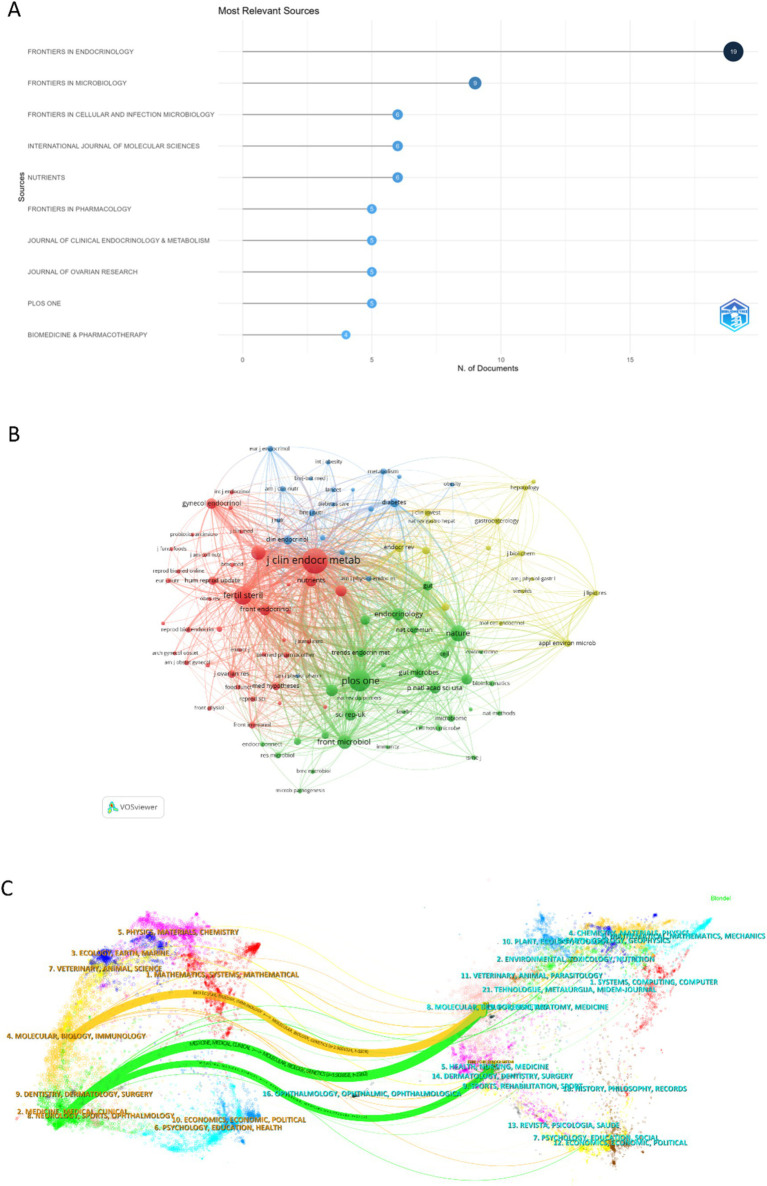
The published journal, co-cited journal, and dual-map overlay of journals research on the field of gut microbiome in PCOS. **(A)** The top 10 published journals in the field. **(B)** The co-citations journals on the gut microbiome in PCOS. **(C)** The dual-map overlay of journals on gut microbiota in PCOS was carried out by Citespace.

The bibliometric analysis revealed that 105 journals collectively published 191 articles on the gut microbiome in PCOS. The journals with the highest publication volumes were Frontiers in Endocrinology, followed by Frontiers in Microbiology, Frontiers in Cellular and Infection Microbiology, International Journal of Molecular Sciences, and Nutrients (Figure 5A). As illustrated in Table 4, the impact factors of the top 10 journals ranged from 2.9 to 6.9, with the majority exceeding 4. Notably, most of these journals are headquartered in the United States.

**Table 4:** Top 10 journals with the most publications on the gut microbiome in PCOS.

Rank	Journals	Publication	Impact factor(2023)a	JCR region	Country of publication
1	Frontiers in endocrinology	19	3.9	Q2	USA
2	Frontiers in microbiology	9	4.0	Q2	Switzerland
3	Frontiers in cellular and infection microbiology	6	4.6	Q1	USA
4	International journal of molecular sciences	6	4.9	Q1	USA
5	Nutrients	6	4.8	Q1	Switzerland
6	Frontiers in pharmacology	5	4.4	Q1	Switzerland
7	Journal of clinical endocrinology & metabolism	5	5.0	Q1	USA
8	Journal of Ovarian Research	5	3.8	Q1	England
9	Plos one	5	2.9	Q1	USA
10	Biomedicine & pharmacotherapy	4	6.9	Q1	France

a: Impact factor.in the category according to Journal Citation Reports (2023).

In this study, we identified a total of 2,051 co-cited journals. Following the implementation of a screening process that employed a minimum co-citation threshold of 27, a subset of 105 co-cited journals was selected for the construction of the co-citation network. The Journal of Clinical Endocrinology & Metabolism exhibits extensive co-citation relationships with numerous journals, including PLOS ONE, Fertility and Sterility, Frontiers in Microbiology, Nature Medicine, and Nature (Figure 5B).

Figure 5C presents a dual-map overlay illustrating citation links between journals and their co-cited counterparts concerning the gut microbiome in PCOS. This overlay offers a clear view of both broad scientific fields and specific specialties, allowing for a quick understanding of each field’s key areas and knowledge exchange (Zhu et al., 2024). The left clusters in Figure 5C represent the source journals, and the right clusters represent the citing journals. Orange and green pathways highlight key citation trends: research in molecular/biology/immunology fields is mainly cited by molecular/biology/genetics literature, while research in medicine/medical/clinical fields is primarily cited by molecular/biology/genetics and health/nursing/medicine literature.

### 3.6 Co-cited reference

**Figure 6:**
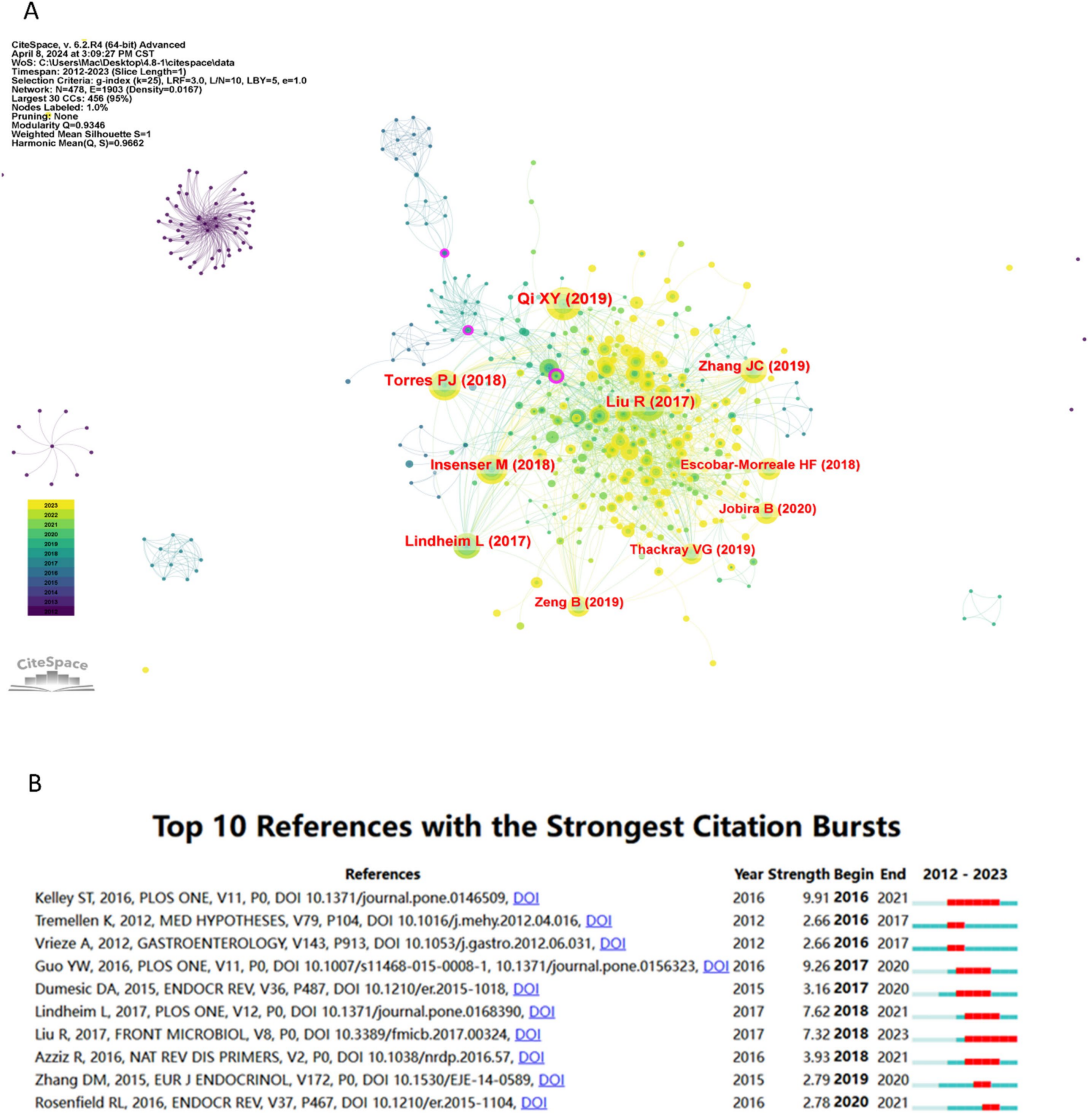
The co-cited references and references with the strongest citation bursts. **(A)** The top 10 frequent co-cited references, and the larger the circle, the more frequently it has been co-cited. **(B)** The top 10 references with the strongest citation bursts.

As illustrated in Figure 6A, the co-citation reference network comprises 478 nodes and 1,903 edges, with a Modularity Q value of 0.9346 and a Mean Silhouette score of 1, thereby satisfying the analytical requirements. The 10 most frequently co-cited references are also depicted in Figure 6A. The highest-ranked co-cited reference is the article titled “Gut microbiota-bile acid-interleukin-22 axis orchestrates polycystic ovary syndrome” by Qi XY, published in Nature Medicine in 2019. This study by Qi XY investigates the role of the gut microbiome and metabolic pathways in regulating ovarian dysfunction and insulin resistance associated with PCOS (Qi et al., 2019).

Figure 6B illustrates the top 10 references with the strongest citation bursts. These references exhibit burst strengths ranging from 2.66 to 9.91, with durations of influence extending from 2 to 6 years. The reference with the highest citation burst was published in PLOS ONE in 2016, with a burst strength of 9.91, spanning from 2016 to 2021. This study indicated that gut dysbiosis occurs in women with PCOS, particularly those with hyperandrogenemia, independent of dietary influences (Kelley et al., 2016). The latest burst citation reference is published by Liu R, and bursting from 2018 to 2023, with a burst strength of 7.32. The article focused on the relationship between gut microbial dysbiosis and PCOS disease phenotypes (Liu et al., 2017).

### 3.7 Keyword analysis

**Figure 7:**
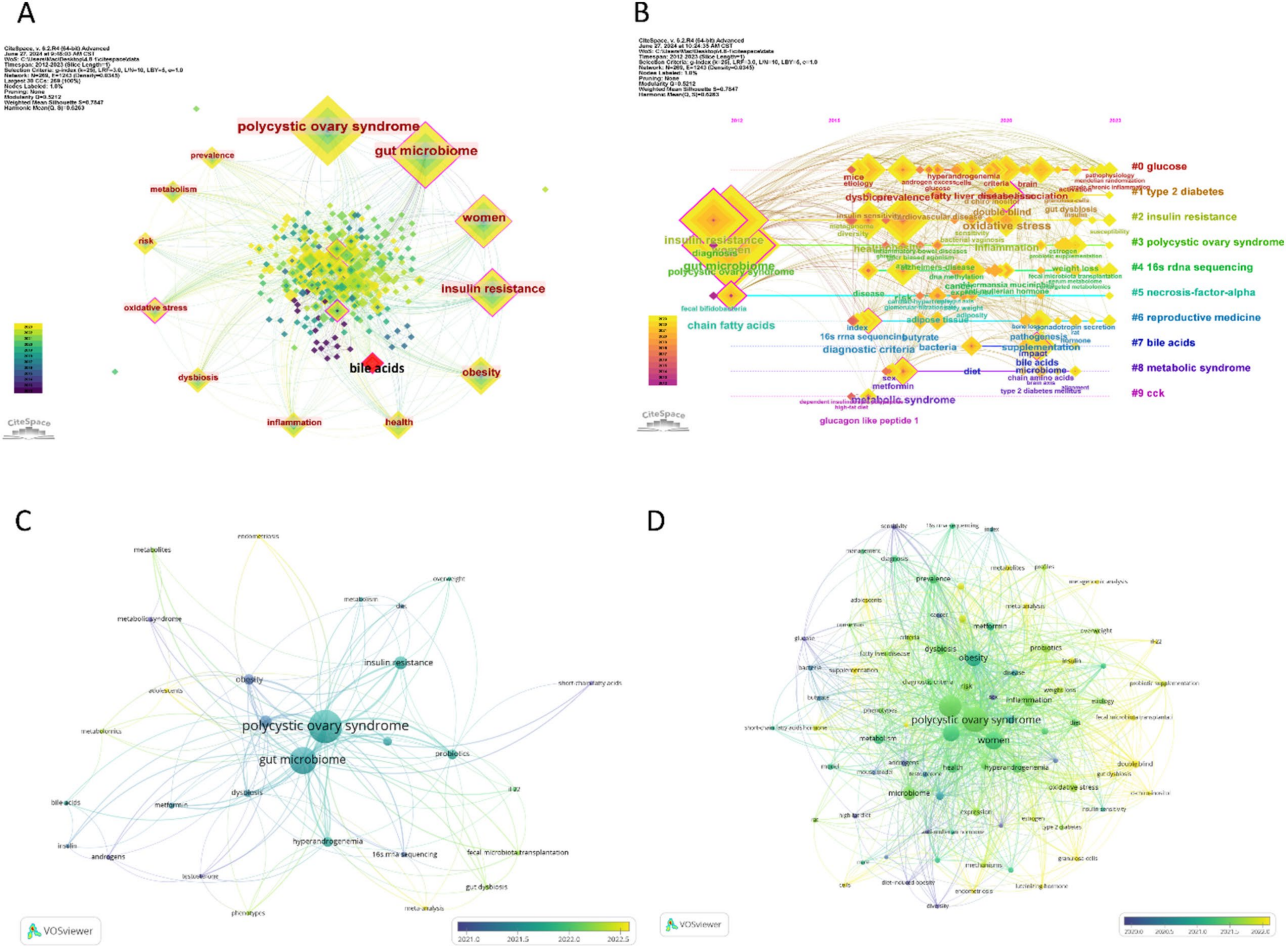
Visualization of keywords and trend topics on the gut microbiome in PCOS, and the larger the square or circle, the higher the publication frequency. **(A)** The top 10 frequency keywords in the field, and the red square represent the strongest citation bursts keyword. **(B)** The clusters of keywords. **(C)** The overlay visualization of author keywords. **(D)** The overlay visualization of all keywords.

Utilizing CiteSpace to analyze the keyword frequency and keyword clusters related to the gut microbiome in PCOS, the timeline view analysis yielded a Modularity Q score of 0.5212 and a Mean Silhouette score of 0.7847, thereby satisfying the analytical requirements. Nineteen keywords appear more than 10 times, and 33 keywords appear more than 5 times. The top twelve frequency keywords of the gut microbiome in PCOS were as follows: polycyclic ovary syndrome (113 times), gut microbiome (116 times), women (74 times), insulin resistance (59 times), obesity (40 times), health (24 times), inflammation (18 times), dysbiosis (18 times), oxidative stress (16 times), risk (15 times), metabolism (15 times), and prevalence (15 times) (Figure 7A). The keyword “bile acids” demonstrated the most pronounced citation bursts, with a burst strength of 2.71, making it the sole keyword to exceed a burst strength of 2 during the period from 2021 to 2023. The timeline view, as depicted in Figure 7B, illustrates the top 10 largest keyword clusters, including #0 glucose, #1 type 2 diabetes, #2 insulin resistance, #3 polycyclic ovary syndrome, #4 16 s rDNA sequencing, #5 necrosis-factor-alpha, #6 reproductive medicine, #7 bile acids, #8 metabolic syndrome, #9 cck.

In our analysis using VOSviewer, we set the threshold to 4, integrated synonyms, and excluded non-essential keywords to examine the “Author Keywords” and “All Keywords” within the “Co-occurrence” framework, employing overlay visualization. This process identified 29 “Author Keywords” and 84 “All Keywords” that satisfied the criteria. Among these, the top five keywords with the highest frequency of occurrence were: for “Author Keywords”—polycystic ovary syndrome (114 occurrences), gut microbiome (96 occurrences), insulin resistance (26 occurrences), microbiome (13 occurrences), obesity (19 occurrences); and for “All Keywords”—polycystic ovary syndrome (139 occurrences), gut microbiome (119 occurrences), women (79 occurrences), insulin resistance (62 occurrences), obesity (57 occurrences).

As Figure 7C shows, the author keywords of the gut microbiome in PCOS, such as gut dysbiosis, phenotypes, adolescents, metabolomics, metabolites, meta-analysis, endometriosis, fecal microbiota transplantation, IL-22, occurred recently, indicating the current hotspots or frontiers in the field. Similarly, all keywords mainly include granulosa-cell, insulin, gut dysbiosis, adolescents, meta-analysis, metagenomic analysis, probiotic supplantation, fecal microbiota transplantation, metabolites, IL-22, granulosa-cells, endometriosis, luteinizing-hormone, d-chiro-inositol, and mechanisms (Figure 7D), which represent the current hotspots or frontiers in the field. This implies that the latest research focuses on the pathogenesis of PCOS connecting with the gut microbiome, the gut microbiome treatment in PCOS. Table 5 shows the hotspots and research trends of the gut microbiome in PCOS, as identified through the keyword and co-cited reference analysis.

**Table 5:** Hotspots and research trends on the gut microbiome in PCOS.

Hotspots and research trends	Keywords and Keyword clusters	Co-cited references and reference burst strength
How gut dysbiosis affects the pathogenesis of PCOS through endocrine and metabolic pathways.	insulin resistance, insulin, obesity, gut dysbiosis, mechanisms, #1 type 2 diabetes, #2 insulin resistance, #3 polycystic ovary syndrome, #8 metabolic syndrome	The gut microbiome is altered in a letrozole-induced mouse model of polycystic ovary syndrome (Kelley et al., 2016). (burst strength: 9.91) Dysbiosis of gut microbiota (DOGMA)--a novel theory for the development of polycystic ovarian syndrome (Tremellen and Pearce, 2012). (burst strength: 2.66) Scientific statement on the diagnostic criteria, epidemiology, pathophysiology, and molecular genetics of polycystic ovary syndrome (Dumesic et al., 2015). (burst strength: 3.16) Dysbiosis of gut microbiota associated with clinical parameters in polycystic ovary syndrome (Liu et al., 2017). (burst strength: 7.32) The pathogenesis of polycystic ovary syndrome (PCOS): the hypothesis of PCOS as functional ovarian hyperandrogenism revisited (Rosenfield and Ehrmann, 2016). (burst strength: 2.78) Polycystic ovary syndrome (Azziz et al., 2016). (burst strength: 3.93)
The biomarkers of inflammation and metabolites of gut microbiome in PCOS.	SCFA, bile acid, IL-22, metabolites, inflammation, oxidative stress, #0 glucose, #5 necrosis-factor-alpha, #7 bile acids	Alterations in gut microbiome composition and barrier function are associated with reproductive and metabolic defects in women with polycystic ovary syndrome (PCOS): a pilot study (Lindheim et al., 2017). (burst strength: 7.62) Serum zonulin is elevated in women with polycystic ovary syndrome and correlates with insulin resistance and severity of anovulation (Zhang et al., 2015) (burst strength: 2.79)
The frontier research in the gut microbiome treatment of PCOS.	fecal microbiota transplantation, probiotic supplantation, bile acid, IL-22, adolescents	Transfer of intestinal microbiota from lean donors increases insulin sensitivity in individuals with metabolic syndrome (Vrieze et al., 2012). (burst strength: 2.66)

## 4 Discussion

### 4.1 The main finding

In this study, we employed a range of bibliometric software tools, including Citespace, VOSviewer, and Bibliometric R, to conduct a comprehensive objective visualization analysis of the development of research on the gut microbiome in PCOS over the past 12 years. By utilizing these software tools to examine publication trends, geographical distribution, institutional affiliations, authorship, co-cited authors, journals, co-cited journals, co-cited references, and keywords within this field, we identified a notable increase in the overall trend of publications in recent years. The number of publications on the gut microbiome in PCOS has shown a yearly increase from 2018 to 2023, reaching its peak in 2023, albeit with fewer than 60 publications. This trend suggests that research in this area is still in its nascent stages. China emerged as the leading country in terms of the number of publications, followed by the USA, India, Italy, and Türkiye. Notably, China and the USA exhibited strong collaborative efforts. Such robust cooperation and exchange are advantageous for the advancement of future research on the gut microbiome in PCOS. Among the top 10 institutions with the highest number of publications in the field, eight were from China and two from the USA. Shanghai Jiao Tong University had the most publications. Peking University, the University of California San Diego, and San Diego State University were the top three total citation institutions.

In the top 10 publishments authors, Thackray VG and Kelley ST had the most output and H- index in the field. It implies that those two authors make important contributions to the research of the gut microbiome in PCOS. They ever focused on hyperandrogenism PCOS (Kelley et al., 2016; Torres et al., 2018; Ho et al., 2021), and found that dysbiosis of the gut microbiome may play a causal role in PCOS (Torres et al., 2019), and research into small-molecule control of gut microbial diversity and host physiology may provide new therapeutic options for the treatment of PCOS (Ho et al., 2021). The most co-cited author was Liu R, whose article had the latest burst citation, and their team found that gut microbial dysbiosis in women with PCOS is associated with the disease phenotypes (Liu et al., 2017). The top co-cited reference is Qi XY’s 2019 article in Nature Medicine, “Gut microbiota-bile acid-interleukin-22 axis orchestrates polycystic ovary syndrome.” They suggest that modifications to the gut microbiome, alterations in bile acid metabolism, and/or enhancements in interleukin-22 (IL-22) levels may be advantageous for the treatment of PCOS (Qi et al., 2019).

The top three publishment journals were Frontiers in Endocrinology, Frontiers in Microbiology, and Frontiers in Cellular and Infection Microbiology. Most of the top 10 journals came from the USA, and the impact factors were 2.9–6.9. The Journal of Clinical Endocrinology & Metabolism emerged as the most frequently co-cited journal, focusing on the disorder of endocrine and metabolism, and exhibiting extensive co-citation relationships with numerous other academic journals. The dual-map overlay of journals reflects the knowledge diffusion path from basic theory to the applied field (Zhu et al., 2024). Current research on the gut microbiota in PCOS mainly sources from molecular/biology/immunology and medicine/medical/clinical fields, and is cited by molecular/biology/genetics and health/nursing/medicine fields. It reflects the knowledge diffusion path from basic theory to applied field in the study of the gut microbiota in PCOS.

### 4.2 Identification of trend and future of evidence synthesis

The analysis of keyword frequency and clusters highlights the dominant topics and research trends in the field. Utilizing Citespace, the study identified five distinct hot topics related to the gut microbiome in PCOS. The foremost topic pertains to endocrine and metabolic disorders associated with the gut microbiome in PCOS, encompassing keyword clusters such as #1 type 2 diabetes, #2 insulin resistance, #3 polycystic ovary syndrome, #8 metabolic syndrome, and most of the top 12 frequently occurring keywords within this domain. Additional significant topics include biomarkers of inflammation and metabolites (#0 glucose, #5 necrosis-factor-alpha, #7 bile acids), microbiome research (#4 16 s rDNA sequencing), reproductive medicine (#6 reproductive medicine), and the modulation of the digestive system (#9 cck). The VOSviewer analysis indicates that recent research focuses on the pathogenesis of PCOS concerning the gut microbiome, as well as the gut microbiome treatment in PCOS, and that adolescents with PCOS should pay more attention to. The latest keywords include “endometriosis” and show that except PCOS, the relationship between gut microbiome and female infertility disorders within the field of reproductive medicine, such as endometriosis, may also warrant increased attention recently (Chadchan et al., 2022).

The etiology and pathogenesis of PCOS are increasingly linked to the gut microbiome, highlighting the significance of gut dysbiosis in influencing the endocrine and metabolic disturbances associated with PCOS, including insulin resistance, obesity, hormonal imbalances, and inflammation (Lindheim et al., 2017; Insenser et al., 2018; Thackray, 2019; Yurtdas and Akdevelioglu, 2020; Kumari et al., 2024). Firstly, insulin resistance is one of the core pathological mechanisms of PCOS (Dunaif, 1997). Recently research focused on insulin resistance in the gut microbiome and PCOS field. Gut disorders change the pathway of metabolite and the inflammatory response, leading to insulin resistance (Gomes et al., 2018). PCOS patients with insulin resistance show an increased abundance of Enterococcus, and the insulin resistance pathway may impact the host environment, contributing to the occurrence and development of PCOS (He and Li, 2021). Secondly, the gut microbiome may affect hormone balance in the body through the gut-brain axis and the gut microbiota-ovary axis (Insenser et al., 2018; Armstrong et al., 2023; Xu et al., 2023). Hyperandrogenemia is one of the characteristics of PCOS. The hyperandrogenemia observe in PCOS may significantly alter the gut microbiome independently of diet, and the steroid hormone levels may regulate the composition of the gut microbial community and metabolism (Kelley et al., 2016), although the relationship between hyperandrogenism in the gut microbiome and PCOS is still unclear (Giampaolino et al., 2021). Women with PCOS show lower *α* diversity than healthy women, and hyperandrogenism is linked to *β* diversity (Torres et al., 2018). Those with only polycystic ovarian morphology have α diversity levels between healthy women and POCS patients (Torres et al., 2018). This suggests that studying the gut microflora differences between polycystic ovarian morphology and PCOS is important for further research. In PCOS patients, Bacteroides, Escherichia/Shigella, and Streptococcus levels are increased and positively correlated with testosterone and body mass index (Liu et al., 2017). Gut dysbiosis may be a factor in PCOS with obesity, with obesity playing a driving role in the development of dysbiotic gut microbiota in PCOS (Liang et al., 2020). Third, Gut dysbiosis in women with PCOS increases gut permeability, allowing lipopolysaccharides from Gram-negative bacteria to enter the bloodstream, which activates the immune system and raises proinflammatory cytokines, causing chronic inflammation (Tremellen and Pearce, 2012; Qi et al., 2019). At the same time, these cytokines disrupt insulin receptor function, causing insulin resistance and hyperinsulinemia. Gastrointestinal hormones like Ghrelin, peptide YY, bile acids, IL-22, and *Bacteroides vulgatus* also play a significant role (Giampaolino et al., 2021).

Intestinal metabolites are important mediators in the occurrence of PCOS. The disorder of the gut microbiome metabolite through many pathways affects PCOS, and gut microbiome metabolite includes carbohydrates, bile acids, short-chain fatty acid (SCFA), branched-chain amino acids, lipopolysaccharide, and gut-brain axis (Cree-Green et al., 2019; Chang et al., 2021; Ho et al., 2021; Tayachew et al., 2022; Wu et al., 2022; Liu et al., 2023; Mukherjee et al., 2023). According to the keyword clusters and keyword citation burst, the metabolisms of bile acids and SCFA pathways have been paid much more attention recently. The gut microbiome affects the combination of bile acids, similarly, bile acids influence the gut microbiome composition (Guo et al., 2022). In PCOS patients, primary bile acids were significantly and positively associated with serum concentrations of total testosterone and androstenedione. Additionally, increased circulating conjugated primary bile acids are positively associated with hyperandrogenism in women with PCOS (Zhang B. et al., 2019). Furthermore, bile acids have been shown to modulate the function of ovarian cells and are implicated in the pathogenesis of PCOS (Yang et al., 2021). Bile acids are linked to IL-22 levels, more *Bacteroides vulgatus*, and lower glycodeoxycholic and tauroursodeoxycholic acid levels, affecting IL-22 modulation (Qi et al., 2019). This may improve insulin resistance and ovarian dysfunction in PCOS, suggesting a new treatment approach (Qi et al., 2019). The gut microbiome, through fermentation, produces SCFA, which is instrumental in reducing insulin resistance and ameliorating symptoms of PCOS (Salehi et al., 2024). However, it is concerning that the abundance of gut bacteria responsible for SCFA production is lower in women with PCOS compared to the normal control group (Salehi et al., 2024). Specifically, the level of SCFA butyric acid is reduced in obese women with PCOS. Butyric acid has been shown to enhance metabolic function and mitigate the inflammatory response in granulosa cells under inflammatory conditions (Liu et al., 2023). Nevertheless, these findings require further validation through clinical trials and multi-omics approaches.

Frontier research focuses on the gut microbiome treatment of PCOS, which mainly includes prebiotics supplantation, probiotics supplantation, and fecal microbiota transplantation (FMT) (Qi et al., 2019; Angoorani et al., 2023; Borzan et al., 2023; Salehi et al., 2024). A potential future therapeutic strategy for PCOS could include the administration of IL-22 and the bile acid glycodeoxycholic acid (Giampaolino et al., 2021). Those new treatments may solve the basic pathogenic mechanisms of PCOS, which play effective roles in anti-inflammatory, insulin-sensitizing, and anti-obesity activities, and relate to the gut microbiome (Bashir et al., 2022). The efficacy of probiotic supplementation in ameliorating the condition of women with PCOS has been well-documented. Regular intake of probiotics has been shown to correct dysbiosis of the gut microbiome and enhance the production of SCFA, which contributes to the reduction of insulin resistance and the alleviation of PCOS symptoms (Salehi et al., 2024). The administration of prebiotics and probiotics in women diagnosed with PCOS has been shown to enhance lipid metabolism, as well as hormonal parameters, including the Free Androgen Index and sex hormone-binding globulin, and inflammatory markers such as nitric oxide and malondialdehyde (Shamasbi et al., 2020; Giampaolino et al., 2021). What’s more, PCOS might begin early in development and manifest in adolescence, monitoring the microbiome and using probiotics in childhood and adolescence could help prevent PCOS by addressing dysbiosis (Calcaterra et al., 2023). *Escherichia coli* Nissle 1917 is a genetically regulated probiotic that has demonstrated a commendable safety profile in humans and is effective in ameliorating metabolic and immune system disorders within the gut microbiome (Luo et al., 2023), and the probiotic fixed effect in the intestine is critical for the probiotic function (Zhang J. et al., 2019).

Furthermore, interventions involving Lactobacillus transplantation and FMT have demonstrated efficacy in restoring menstrual cyclicity, improving ovarian follicle morphology, promoting the formation of corpora lutea, and reducing levels of testosterone and androstenedione (Dabke et al., 2019). Metabolic improvements were observed in FMT-treated PCOS rats compared to the untreated group, characterized by reduced androgen levels, increased estradiol and estrone, and normalization of ovarian function (Guo et al., 2016). Compared with prebiotics and probiotics supplantation, FMT is considered to be the most comprehensive treatment. Because FMT not only includes bacteriomes but also viromes, fungi, archaeometry, and even parasites (Corrie et al., 2021). However, most research on FMT in PCOS uses murine models presently.

## 5 Limitations

The data sources for our study are limited to the Web of Science Core Collection, thereby excluding a comprehensive representation of the literature on the gut microbiome in the PCOS field. Furthermore, our analysis was restricted to English-language publications, omitting works in other languages. Due to temporal constraints, research published after 2024 was not incorporated. Consequently, recently published literature with limited citations may not have received adequate consideration. Our research explores the hotspots and research trends of the gut microbiome in PCOS from a macro perspective; however, it does not directly determine causation between the gut microbiome and PCOS.

## 6 Conclusion

This bibliometric analysis identifies the key topics and research trends related to the gut microbiome in PCOS. The analysis reveals a consistent annual increase in publication volume. The most influential countries were China and the USA, with the highest number of publications coming from Shanghai Jiao Tong University. The hottest topic revolves around how gut microbiome disorders influence endocrine and metabolic pathways leading to PCOS. Frontier research focuses on the pathogenesis and treatment of PCOS related to the gut microbiome.

## Data Availability

Publicly available datasets were analyzed in this study. This data can be found at: https://www.webofscience.com/wos/woscc/summary/51aa7501-3651-49af-b53e-ece7e68b78eb-01127bbbfe/date-ascending/1.
