# Supplementary material for: Hotspots and research trends of gut microbiome in polycystic ovary syndrome: a bibliometric analysis (2012–2023)
Source: Front Microbiol. 2025 Jan 8;15:1524521. doi: 10.3389/fmicb.2024.1524521 (PMC11753182; doi:10.3389/fmicb.2024.1524521)
Supplement: Supplementary file 1 [file Table_1.docx]

Supplementary Material

Supplementary information 1

The keywords searching were used: (“Polycystic Ovary Syndrome” OR “Ovary Syndrome, Polycystic” OR ”Syndrome, Polycystic Ovary” OR “Stein-Leventhal Syndrome” OR” Stein Leventhal Syndrome” OR ”Syndrome, Stein-Leventhal” OR ” Sclerocystic Ovarian Degeneration” OR ”Ovarian Degeneration, Sclerocystic” OR “Sclerocystic Ovary Syndrome” OR “Polycystic Ovarian Syndrome” OR “Ovarian Syndrome, Polycystic” OR “Polycystic Ovary Syndrome 1” OR “Sclerocystic Ovaries” OR “Ovary, Sclerocystic” OR “Sclerocystic Ovary”) AND (“Gastrointestinal Microbiomes” OR “Microbiome, Gastrointestinal” OR “Gut Microbiome” OR “Gut Microbiomes” OR “Microbiome, Gut” OR “Gut Microflora” OR “Microflora, Gut” OR “Gut Microbiota” OR “Gut Microbiotas” OR “Microbiota, Gut” OR “Gastrointestinal Flora” OR “Flora, Gastrointestinal” OR “Gut Flora” OR “Flora, Gut” OR “Gastrointestinal Microbiota” OR “Gastrointestinal Microbiotas” OR “Microbiota, Gastrointestinal” OR “Gastrointestinal Microbial Communit*” OR “Gastrointestinal Microbial Communiti*” OR “Microbial Community, Gastrointestinal” OR “Gastrointestinal Microflora” OR “Microflora, Gastrointestinal” OR “Gastric Microbio*” OR “Microbiome, Gastric” OR “Intestinal Microbio*” OR “Microbiome, Intestinal” OR “Intestinal Microbiota*” OR “Microbiota, Intestinal” OR “Intestinal Microflora” OR “Microflora, Intestinal” OR “Intestinal Flora” OR “Flora, Intestinal” OR “Enteric Bacteria” OR “Bacteria, Enteric”).
